# The Effect of Different Sources of Selenium Supplementation on the Meat Quality Traits of Young Charolaise Bulls during the Finishing Phase

**DOI:** 10.3390/antiox10040596

**Published:** 2021-04-13

**Authors:** Silvia Grossi, Luciana Rossi, Michele De Marco, Carlo Angelo Sgoifo Rossi

**Affiliations:** 1 Department of Animal Science and Food Safety, University of Milan, Via dell’Università 1, 26900 Lodi, Italy; silvia.grossi1994@libero.it (S.G.); luciana.rossi@unimi.it (L.R.); carlo.sgoifo@unimi.it (C.A.S.R.); 2 Adisseo France SAS, Immeuble Antony Parc II, 10 Place du Général de Gaulle, 92160 Antony, France

**Keywords:** beef cattle, meat quality, sodium selenite, selenium yeast, hydroxy-selenomethionine

## Abstract

The aim of the study was to compare the effects of sodium selenite (SS), selenium yeast (SY), and hydroxy-selenomethionine (OH-SeMet) on the meat quality and selenium (Se) deposition of finishing beef cattle. Sixty-three bulls were distributed over 3 treatments and fed SS, SY, or OH-SeMet at 0.2 mg kg^−1^ dry matter (DM) for 60 d. None of the Se sources affected the growth performance or carcass characteristics. OH-SeMet showed a higher Se transfer to the meat than SS or SY (*p* < 0.01). SY and OH-SeMet reduced the shear force of the meat (*p* < 0.0001), improved pH (*p* < 0.001), and reduced the drip losses (*p* < 0.001) and the lipid oxidation of the meat (*p* < 0.001). During 8 d of storage, OH-SeMet showed higher levels of meat lightness (L*) and yellowness (b*) than SS (*p* < 0.001), while the SY meat showed a higher L* than SS, albeit only on d 6. OH-SeMet improved b*, compared to SS, and also compared to SY on days 4, 7, and 8 (*p* < 0.001). Supplementing beef with SY and OH-SeMet improved several meat quality parameters. OH-SeMet appears to be the most effective strategy to improve the Se content and color stability of beef cattle meat.

## 1. Introduction

In most developed countries, human nutritional practices have recently changed their focus from fighting nutrient deficiencies to addressing nutrient requirements and food quality to maintain good health throughout life. Therefore, “quality” and “health” are becoming two of the most important factors that influence the food choice of consumers [1]. In this regard, the appearance, color, texture, taste, aroma, and organoleptic composition of meat are the key quality attributes that affect the acceptance of meat by consumers [2].

Lipids are important components of all types of meat and are responsible for their desirable characteristics; however, lipids are susceptible to degradation [3]. The development of oxidative rancidity in meat begins at slaughtering, and it continues progressively during its handling, processing, storage, and cooking. Moreover, lipid oxidation is the main non-microbial cause of a quality deterioration of meat and meat products [3,4]. This process leads to discoloration, drip and cooking losses, a loss of nutritional value, off-odor and off-flavor development, texture defects, and the production of potentially toxic compounds, all of which are important reasons for consumers to reject meat [5,6]. In this context, appropriate animal nutrition practices should be adopted to match the production goals of modern livestock genetics and increase the quality of the final products to meet the increasing market requests for quality and healthy meat.

It is known that the presence of exogenous antioxidants in animal diets can increase the stability of the lipids and proteins in meat [7,8]. Among the antioxidants that can be used in animal feeds, selenium (Se) is one of the main antioxidants used as a dietary supplement, together with vitamin E, to control lipid and protein oxidation processes, as it has the ability to improve both the quality characteristics of meat and also its nutritional value, in particular in terms of Se content [6,9].

The biological functions of Se are tackled by 25 selenoproteins, which have many metabolic functions, and more than half of these are directly or indirectly involved in the maintenance of the body redox balance and antioxidant defense [10,11,12]. Therefore, an adequate feed supplementation with Se plays a pivotal role in decreasing the risk of immunodeficiency, maintaining animal performances, and preserving the quality of the final products, such as meat [13]. In this regard, in order to avoid Se deficiency and to fulfil the Se requirements of livestock animals, feeds are always supplemented with Se in inorganic form (sodium selenite, SS; blends of SS and soya protein hydrolysates), or in organic form. One of the organic forms, Se-yeast (SY, organic forms of Se synthesized by yeast), has been authorized as a feed supplement to provide at least >63% of the total Se as selenomethionine (SeMet) while, more recently, pure chemically synthesized SeMet forms, such as hydroxy-selenomethionine (OH-SeMet), have been authorized as a feed supplement to provide >98% of the total Se as OH-SeMet [14,15,16,17].

The main advantage of feeding animals Se in the form of SeMet over inorganic sources or other organic Se compounds (e.g., dietary forms of selenocysteine) is that SeMet is metabolized as a constituent of the methionine pool and this leads to a storage depot of Se being created in the body tissues of animals [12,13]. For this reason, the use of organic Se in the form of SeMet has been shown to be the best strategy to produce Se-enriched foods, such as eggs [18], milk [19,20,21], and meat [22,23,24,25], as a way of preventing Se deficiency in human diets [26]. To date, the functional benefits of OH-SeMet, compared to both SS and SY, have been described for broiler chickens [27], laying hens [18], pigs [28], and dairy cattle [20], but not for beef cattle.

Considering the lack of studies regarding the effect of OH-SeMet on beef cattle, the aim of the present study has been to evaluate its effects on the growth performance, carcass characteristics, and meat quality of Charolaise bulls, and the Se content of the meat, compared to SS or SY, administered to reach 0.2 mg Se kg^−1^ dry matter of feed, during the last 60 days of the finishing period.

## 2. Materials and Methods

### 2.1. Study Site

The study was performed on a commercial intensive beef-fattening farm located in the northeast of Italy. The procedures involving the animals were carried out in accordance with the European Community Council Directive (2010/63/EU), transposed by the Italian Ministry of Health (Legislative Decree 26, 4 March 2014). The study protocol was approved by the Animal Welfare Organization (OPBA), established by the Rector of the University of Milan, Italy (ethical code: 3187559) on 31 July 2020.

### 2.2. Animals, Experimental Design, and Treatments

A total of 63 Charolaise bulls, with an average age of 521 ± 32 days, were used for the study. The animals were randomly distributed into 3 dietary treatments with 3 slatted floor pen replicates of 7 bulls each (average body weight: 641 ± 12 kg). The bulls were fed the same total mixed ration (TMR, Table 1), which was formulated to satisfy the National Research Council (NRC) nutritional requirements [29], administered ad libitum and delivered once a day in the morning, by means of a feed mixer wagon, provided with electronic scales to weigh the inclusion of each ingredient and the unloaded TMR. The background Se concentration in the TMR was 0.138 mg/kg on a dry matter (DM) basis. Water was available ad libitum.

Only the Se source was different in the three experimental groups, that is, sodium selenite (SS), Se-enriched yeast (SY) and hydroxy-selenomethionine (OH-SeMet), respectively. The three different Se sources were administered through the mineral and vitamin premix, and their inclusion was targeted to provide 0.2 mg Se kg^−1^ dry matter of feed. The trial was conducted over the last 60 days prior to slaughtering.

### 2.3. Growth Performance and Health Status

The individual body weight (BW) was recorded, prior to the morning feeding, on enrolment day (day 0) and the day before slaughtering (day 60), and the average daily gain (ADG) was subsequently calculated. The health status of the animals was monitored daily by a veterinarian and no health problems were recorded.

### 2.4. Meat Samples and Analysis

At the end of the finishing period, all the animals were slaughtered at the same slaughterhouse and the carcass characteristics were evaluated. The carcass evaluation was assessed by an expert judge, according to EU legislation [30], using the SEUROP classification method, with a conformation scale ranging from S to P (S—superior: all profiles extremely convex, exceptional muscle development, double-muscled conformation; E—excellent: all profiles convex to super-convex, exceptional muscle development; U—very good: profiles on the whole convex, very good muscle development; R—good: profiles on the whole straight, good muscle development; O—quite good: profiles straight to concave, medium muscle development; P—poor: all profiles concave to very concave, poor muscle development), and with a fatness scale ranging from 1 to 5 (1—low: none up to low fat cover; 2—slight: slight fat cover, flesh visible almost everywhere; 3—medium important: flesh, with the exception of the round and shoulder, almost everywhere covered by fat, slight fat deposits in the thoracic cavity; 4—high: flesh covered by fat, round and shoulder still partly visible, medium fat deposits in the thoracic cavity; 5—very high: carcass well covered by fat, heavy fat deposits in the thoracic cavity).

The cold carcass weight was obtained after 48 h of chilling at a temperature of 0 °C to 4 °C. At the same time, samples of *Longissimus thoracis* muscle, taken between the 5th and the 7th rib, were removed from 10 homogenous carcasses from each experimental group.

Each sample was divided into three 2.50 cm thick subsamples. Two samples that had been stored for 8 days in a plastic box, wrapped with polyethylene film, and kept at 0 °C to 4 °C in a dark room, were used to evaluate the meat color, pH, water holding capacity (WHC), shelf-life and, at the end of the display period, lipid oxidation. The third sub-sample was weighed, vacuum-packaged, and kept frozen at −20 °C until chemical and physical analysis.

The pH was measured by means of a portable pH-meter (HI 98150, HANNA Instruments Inc., Woonsocket, RI, The USA) equipped with a glass electrode (3 mm Ø conic tip) suitable for meat penetration, and values were obtained for each sample from the average of three measurements. The color analysis was performed, using a CR310 Chromameter, on an 8-mm measuring area (Konica Minolta Sensing Americas, Inc, Ramsey, NJ, USA). The chromameter was set on D65 illuminant and calibrated with the CIELab color space system using a white calibration plate (Calibration Plate CR-A43, Konica Minolta Sensing Americas, Inc, Ramsey, NJ, USA), according to the CIELab system. Lightness (L*), redness (a*) yellowness (b*), hue angle (h), and chroma (C) values were calculated for each sample as the average of 10 repetitions. The samples were blot dried and weighed daily, and the steak was placed on the weight scales from the opposite side to that used for color determination; the drip losses were calculated as the sum of the weight lost during the 8 days of storage. The chemical composition (dry matter, ether extract, crude proteins, and ashes) was determined on samples trimmed of external fat and connective tissue, and homogenized for 30 s, according to Association of Official Analytical Chemists (AOAC) [31]. The thawing loss was assessed by weighing the frozen samples and again after 24 h of thawing at 4 °C. The cooking loss was determined, as described by Honikel [32], as the weight lost after cooking in a water bath, until a core temperature of 75 °C was reached (monitored with a Hanna Instruments HI98840 temperature meter, HANNA Instruments Inc., Woonsocket, RI, USA), and after 24 h of storage at 4 °C. Before being weighed, all the samples were blot dried. The difference between the pre- and post-cooking weights was used to calculate the percentage lost during cooking. After cooking and then cooling for 24 h at 4 °C, six 1.27 cm diameter cylindrical cores were taken from each steak, parallel to the longitudinal orientation of the muscle fibers. Cores were obtained using a handheld coring device, and any cores that were not uniform in diameter or with obvious connective tissue defects were discarded. In order to avoid the hardening that occurs toward the outside of the sample, each core was sheared once in the center (V-shaped cutting blade with a 60-degree angle), with a Warner-Bratzler Shear Force attachment, using an electronic testing machine (Model 4466, Instron Corp., Canton, MA, USA). The crosshead speed was set at 200 mm/min [33]. The peak force (kg/cm^2^) was then recorded.

The Se contents in fresh meat were assessed using ICP-MS methods (Agilent 7500cx, Agilent Technologies, Inc., Santa Clara, CA, USA). Analytical procedures were performed according to the standards [34].

Lipid oxidation was assessed on d1 and d8 of the storage period, by measuring the malondialdehyde concentration (MDA, expressed in mg/kg of muscle) using the thiobarbituric acid reactive substances (TBARS) procedure [35].

### 2.5. Statistical Analysis

The body weight, average daily gain, carcass weight and characteristics, dressing percentage, meat characteristics and Se content, shear force, thawing, drip and cooking losses, and TBARS were analyzed by means of one-way ANOVA (SAS Institute, Inc., Cary, NC, USA) considering the main effect of the treatment. The color parameters, pH and visual evaluation score were analyzed, by means of two-way ANOVA, using a general linear model for repeated measures, considering the effects of the treatment, storage time and their interaction (SAS Institute, Inc., Cary, NC, USA). The significance level was set and discussed for *p* ≤ 0.05.

## 3. Results

### 3.1. Growth Performance and Carcass Characteristics

The growth performance and carcass characteristics recorded during the trial are summarized in Table 2. The bulls considered in this trial were distributed evenly over the three different treatments and all showed a good health status. Neither the performance parameters (ADG and final BW) nor carcass characteristics were affected by the treatments, regardless of the source of Se used (*p* > 0.05).

### 3.2. Chemical Analysis and Quality Assesment of the Meat

The results of the proximal analyses of the meat, Se deposition in the muscle, the thawing loss, drip loss, cooking loss, and shear force of the meat are presented in Table 3. None of the Se sources used in this trial affected the centesimal composition of the meat (*p* > 0.05). The Se analysis results show that supplementation with organic Se sources significantly increased the Se content of the *Longissimus thoracis* muscle compared to the SS-fed group (*p* < 0.01). Moreover, if the two organic Se sources tested in this study are compared, the OH-SeMet-fed group shows a significantly higher Se muscle deposition than the SY-fed group (*p* < 0.01). Both SY and OH-SeMet significantly reduce the meat shear force, compared to SS (*p* < 0.0001) as well as the meat drip loss measured after 8 days of storage (*p* < 0.001), with the OH-SeMet-fed group showing the lowest values. None of the Se sources used in this trial affected the meat thawing and cooking losses (*p* = 0.302 and *p* = 0.836, respectively).

**Figure 1:**
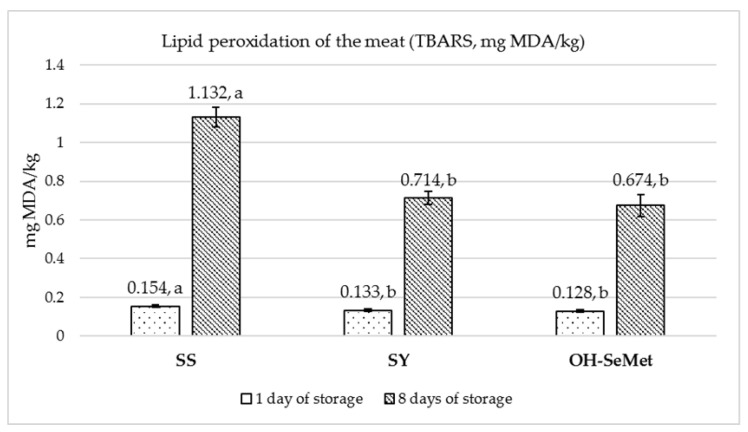
Effect of the selenium sources on the level of thiobarbituric acid reactive substances (TBARS, mg/kg of malondialdehyde—MDA) in the meat during 1 and 8 days of storage. SS—sodium selenite; SY—selenium-enriched yeast; OH-SeMet—hydroxy-selenomethionine. Mean values with dissimilar letters were statistically different (*p* < 0.05). The error bar represents the standard error of the means.

The lipid peroxidation of the meat was evaluated by means of TBARS after 1 and 8 days of storage, as reported in Figure 1. The dietary supplementation of both SY and OH-SeMet significantly reduced the lipid peroxidation of the meat, compared to SS, both after 1 day of storage (*p* < 0.0001) and after 8 days of retail display storage (*p* < 0.0001), with the OH-SeMet group showing the lowest value.

The pH values recorded daily during the 8 days of storage are reported in Table 4. As expected, during storage, the pH of the meat increases (*p* < 0.001), starting from day 4. From day 4 to day 8 of storage, both SY and OH-SeMet maintain significantly lower pH values of the meat than SS (*p* < 0.001).

**Figure 2:**
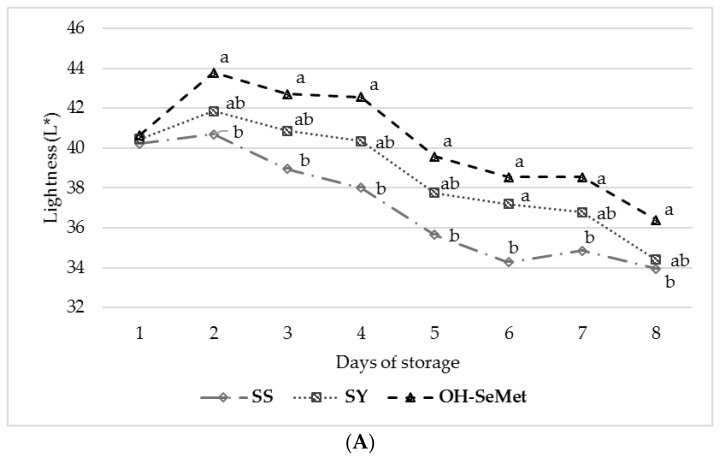

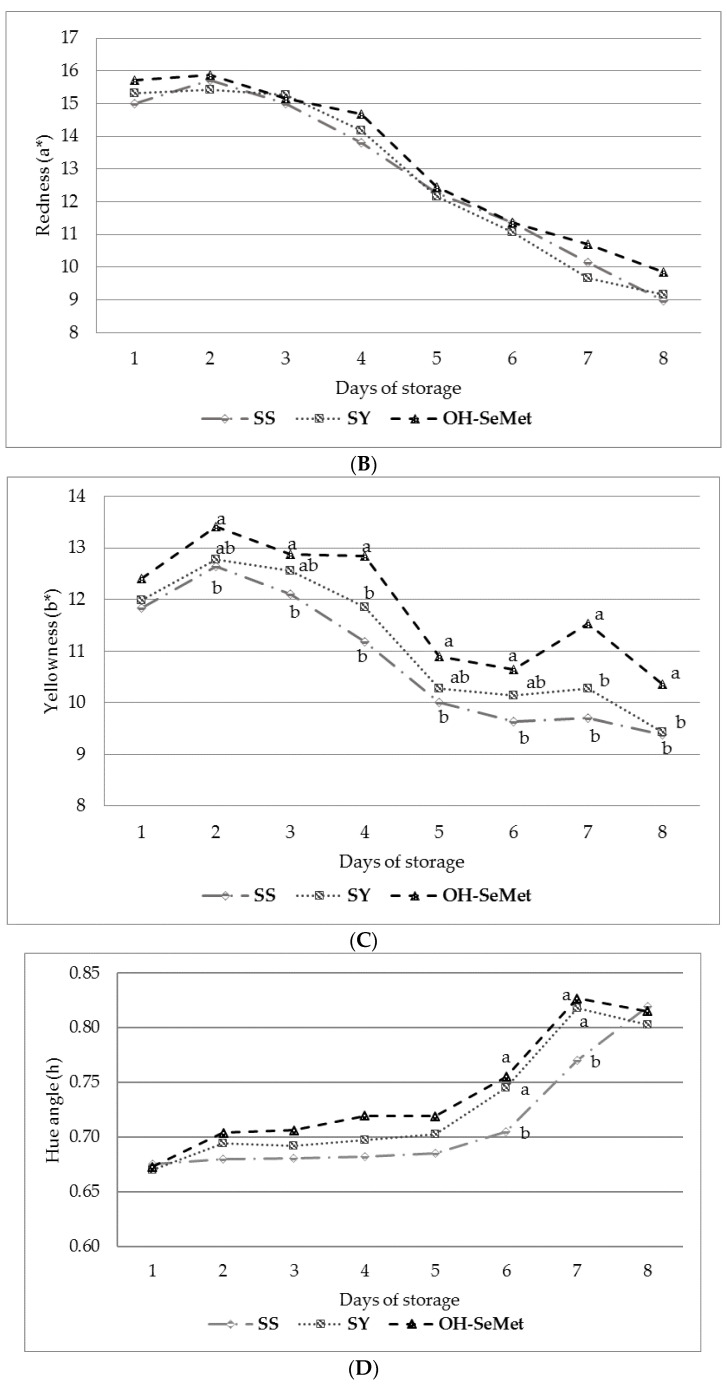

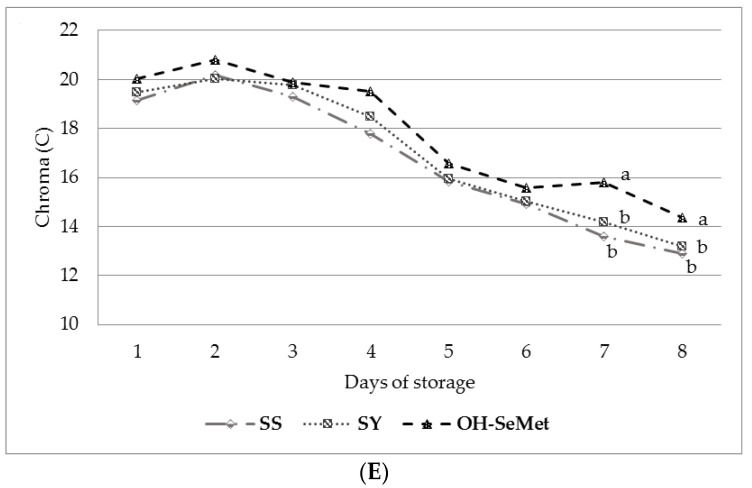
Effect of the selenium source on the lightness (L*) (**A**), redness (a*) (**B**), yellowness (b*) (**C**), hue angle (h) (**D**) and chroma (C) (**E**) of the meat during 8 days of storage. SS—sodium selenite; SY—selenium-enriched yeast; OH-SeMet—hydroxy-selenomethionine. Mean values with different letters are statistically different (*p* < 0.05).

As far as the color parameters are concerned, the lightness of the meat (L*, Figure 2A) decreased over the 8 days of storage, and only the OH-SeMet group showed significantly higher values than SS (*p* < 0.001); SY was intermediate, somewhere between SS and OH-SeMet (*p* > 0.05), and improved the lightness values, compared to SS, albeit only at day 6 of storage. Like lightness, meat redness (a*, Figure 2B) decreased over the 8 days of storage, although none of the Se sources showed an effect on this parameter. The OH-SeMet meat samples showed the highest yellowness values (b*, Figure 2C) over the 8 days of storage and were constantly significantly higher than SS and significantly higher than SY on days 4, 7 and 8 (*p* < 0.001). SY showed a similar yellowness degradation to SS for this parameter (*p* > 0.05). Both SY and OH-SeMet improved the hue angle (h, Figure 2D) of the meat on days 6 and 7 of storage, compared to SS (*p* = 0.0403 and *p* = 0.0146 for SY; *p* = 0.0102 and *p* = 0.0039 for OH-SeMet). The saturation index of the meat (Chroma-C, Figure 2E) decreased over the storage time, but was maintained significantly higher by OH-SeMet on days 7 and 8, compared to both SS and SY, which showed similar values (*p* < 0.001).

## 4. Discussion

The results of the present study indicate no difference in the growth performance or carcass characteristics between treatments, regardless of the Se source supplementation to the diets of beef cattle during the last 60 days prior to slaughtering. These results are in agreement with several other studies carried out on beef cattle [22,24,36,37], calves [38], and lambs [39] and appear to be a logical result, in relation to the short supplemental period and to the absence of major stress applied to the animals during the present study. Given that Se is mainly implicated in improving the antioxidant status and immune response of animals, this result was expected, as the final stage of the fattening cycle is not a critical stage for infectious diseases [13,40]. In fact, it is well known that the benefits of Se, and in particular of organic Se (namely SeMet), deposited in the tissue of animals appear when the animals face specific stressful physiological, environmental, nutritional and/or technological situations [12,40]. Sgoifo Rossi et al. [41] have shown a growth performance improvement in beef cattle supplemented with organic Se, compared to inorganic Se, but after a long-lasting Se supplementation and on younger animals, and this might be explained by the higher bioavailability of organic Se having a positive effect on the health and antioxidant status. Silva et al. [24], comparing SS and SY in an 84-day supplementation study, reported an increased dressing percentage for SY-fed cattle, but when using a much higher Se supplementation level (0.3, 0.9, 2.7 mg Se/kg DM) than those used in the present trial (0.2 mg Se/kg DM).

Similarly, the absence of an effect of Se sources on the centesimal composition of the meat agrees with previously reported data on beef cattle [22,36,42] and lambs [43]. Conversely, other studies have reported a reduction in the meat fat content of beef cattle when fed a super-nutritional level of organic or inorganic Se [37,44]. The rise in the muscular Se concentration achieved in this study, as a result of organic Se in the form of SY or OH-SeMet, compared to SS, is consistent with other studies on beef [22,24,36,42] and lambs [39,43], and this confirms the greater bioavailability of the organic form than the inorganic one. The results of the present study also highlight differences in Se deposition in the muscle between SY and OH-SeMet, with OH-SeMet showing the highest values. To the best of our knowledge, these results represent the first direct comparison between SY and OH-SeMet in beef cattle and confirm the higher bio-efficacy of OH-SeMet previously reported for broiler chickens [27], laying hens [18], pigs [28], and dairy cattle [20]. These findings can be explained by considering the different concentrations of SeMet/OH-SeMet of the two different tested organic Se sources, which were about 63% in the SY and >98% in the OH-SeMet. In this regard, it has recently been confirmed that the differences in between Se additives, in terms of bio-efficacy, are mainly driven by the SeMet content of the additive, and this corroborates the hypothesis that the other Se compounds present in SY (e.g., dietary selenocysteine) do not play significant roles as nutritional sources of Se, in a similarly way to SS [45].

Increasing the Se content of meat is an important outcome, as meat is one of the major contributors to Se intake in the human diet. In most EU countries, the Se intake is lower than the recommended daily allowance of 55 μg Se day^−1^ [26,45]. A low Se status in humans has been linked to an increased risk of developing cancer, cardiovascular disease, thyroid autoimmune disease, problematic fertility/reproduction, rheumatoid arthritis, and a variety of other diseases [26,46], as well as being an important risk factor for viral infection and therapy outcomes [47]. It appears, on the basis of the present results, that the replacement of SS and SY with OH-SeMet would allow selenium-enriched meat to be produced (by maximizing the level of Se of the meat) with a consequently higher added nutritional value.

Color, tenderness, drip loss, and lipid peroxidation are quality aspects that affect the consumers’ willingness to purchase meat products to a great extent. Alterations of these parameters have been related to oxidative stress, defined as an imbalance between antioxidant and prooxidant molecules in the meat, which is able to cause alterations of the pigments, proteins, and lipids [2]. Selenium is of great importance for the antioxidant defense system of cells, as it helps to reduce lipid and protein oxidation and avoid changes in color and undesirable aroma formation [6,9]. In this regard, it has been shown that organic Se, in particular OH-SeMet, enhances glutathione peroxidase, methionine sulfoxide reductase B, and thioredoxin reductase gene expression and activity, as well as the overall total antioxidant capacity [20,48,49]. In this study, the oxidative damage of lipids, as indicated by the high levels of MDA, was significantly reduced after 8 days of storage by both SY and OH-SeMet, compared to SS. These results are in line with those of Silva et al. [24], who reported a reduction in meat MDA levels in beef cattle fed organic Se, compared to inorganic Se. The present findings corroborate the results of another study carried out on pigs, where the SY group showed less MDA in muscle samples after 7 days of refrigerated storage than the SS group [48].

The higher muscle antioxidant capacity achieved by both SY and OH-SeMet also explains the differences observed on meat tenderness, compared to SS. In fact, post-mortem, muscular proteins undergo an oxidation process that could impair enzyme activity, especially the calpains responsible for muscle proteolysis, and increase myofibrillar protein cross-linking [7,50]. Both μ- and m-calpains contain histidine and cysteine thiol groups in their active site, which are highly susceptible to oxidation and a consequent inactivation. Moreover, the cross-linking of myofibrillar proteins reduces their degradation, thereby increasing the strength of the myofibrillar structure and meat toughness. An increase in the meat antioxidant capacity, namely glutathione peroxidase activity, which can be explained in this study by the lower MDA levels recorded for the SY and OH-SeMet, reduces calpain oxidation, thereby helping to preserve the functionality and tenderness of the meat [22]. Our results agree with those of Sgoifo Rossi et al. [22] and Cozzi et al. [42], who also found a reduction in the shear-force of the meat of Charolaise bulls supplemented with organic Se, compared to those fed inorganic Se.

The supplementation of SY and OH-SeMet during the last 60 days of the finishing process led to lower drip losses in the meat after a storage of 8 days at 0 °C to 4 °C than SS. This positive effect of organic Se on beef drip loss is consistent with that observed previously in beef [42], broiler chickens [51], and pigs [25,52]. The effect of organic Se on muscle drip loss may arise from the higher capacity to reduce membrane lipid oxidation and enhance the activation and synthesis of methionine sulfoxide reductase B, a selenoprotein that is able to reduce protein oxidation [12,49]. In the present study, no effects were found for either the thawing or cooking losses for the three different sources of Se. These results agree with those of Cozzi et al. [42], who reported no difference in cooking losses in beef cattle supplemented with either SS or SY.

Regarding the meat pH, our findings show that the pH values gradually increased during the 8 days of storage. After 8 days of storage, the SY and OH-SeMet showed lower pH values than SS but remained within the range of normality. These results are comparable with those reported for beef cattle by Cozzi et al. [42], who, however, showed a statistical trend for SY with higher pH values than SS. In this regard, and due to the limited evidence present in the literature, further investigations are necessary to better clarify the role of Se on the evolution of meat pH during storage.

The color of meat mainly depends on the myoglobin content and muscle oxidation-reduction status [53]. A higher antioxidant defense prevents or reduces and delays oxidation processes of the heme group inside the myoglobin, thereby contrasting its transformation into metmyoglobin. In fact, a greater presence of metmyoglobin than oxymyoglobin leads to darker and duller meat [54]. In the present study, both SY and OH-SeMet maintained a better meat color stability than SS. However, OH-SeMet was found to be the most efficient Se source in maintaining higher meat yellowness, lightness, and chroma values, as it was the only source that constantly showed significantly higher values than SS. These results appear consistent, considering the lower MDA and drip loss values recorded on the same meat sample for SY and OH-SeMet after 8 days of storage. This shows how these two Se forms participate to a great extent in delaying the oxidation processes of meat. In this regard, several authors have found a relationship between lipid and heme-protein oxidation and have shown how lipid and heme-protein oxidation in meat occurs in a concurrent manner and each process appears to enhance the other [4]. The further advantages on meat color stability highlighted in this study for OH-SeMet might be explained by the higher Se level recorded in the meat, which could have provided an extra boost in terms of antioxidant protection, by promoting the gene expression and activity of several selenoproteins. This hypothesis corroborates the finding of Zhao et al. [49], who highlighted how, compared with SS or SY, OH-SeMet demonstrates a unique ability to induce the early expression and enhance the activity of glutathione peroxidase, selenoprotein S, methionine sulfoxide reductase B, and thioredoxin reductase. These specific selenoproteins could participate, through their redox function, in maintaining an optimal muscle function and therefore an optimal meat quality [9,55].

## 5. Conclusions

In conclusion, under the here considered experimental conditions, substituting the Se supplementation of SS with SY and in particular with OH-SeMet during the last 60 days of the fattening period of beef cattle resulted in improved meat tenderness, decreased meat drip losses, and lipid peroxidation, as well as better color stability throughout the storage period. OH-SeMet appears to be much more effective than SY in improving the Se content of meat and in maintaining color stability during storage. These results also demonstrate that a short-term Se supplementation with OH-SeMet represents an effective strategy to create Se enriched meat by improving several quality parameters of the meat at the same time.

## Figures and Tables

**Table 1:** Composition of the diets on a dry matter basis (%).

Item	%
Maize silage	11.00
Corn meal	53.00
Wheat bran	10.00
Soybean meal	4.00
Distillers	7.00
Brewers	4.00
Wheat straw	4.00
Cocoa panel	5.00
Vitamins and minerals ^1^	2.00
**Chemical Characteristics on Dry Matter Basis**	
Dry matter, %	68.41
UFV, units	1.09
MJ	13.13
Crude protein, %	13.18
Sugars, %	3.32
Starch, %	46.18
NDF, %	24.27
Fat, %	4.90
Ca tot, %	0.72
P tot, %	0.48
Se tot, mg/kg	0.138

^1^ Mineral-vitamin premix without any trace minerals: 301.290 international unit (IU) of vitamin A; 35.000 IU of vitamin D3; 1500 mg of vitamin E (all-rac-alpha-tocopheryl acetate); 1.9 mg vitamin E (RRR-alpha-tocopheryl acetate); 19.8 mg of vitamin K; 29 mg of vitamin B1; 16 mg of vitamin B2; 9.8 mg of vitamin B6; 0.15 mg of vitamin B12; 990 mg of vitamin B3; 9 mg of vitamin B5; 0.4 mg of biotin; 38.9 mg of cobalt(II) acetate tetrahydrate; 393 mg of copper sulfate pentahydrate; 39 mg of I; 839 mg of Mg; 2790 mg of Zn; 9.9 mg of selenium (Se) as sodium selenite, selenium-enriched yeast or as hydroxy-selenomethionine—depending on the treatment. UFV: feed unit of maintenance and meat production; MJ: megajoule; NDF: neutral detergent fiber.

**Table 2:** The effect of the selenium source during the finishing phase on the growth performance and carcass characteristics.

Item	Groups			SEM	*p*
	SS	SY	OH-SeMet		
Average initial BW, kg	643.48	640.67	639.33	2.435	0.475
Average final BW, kg	752.86	751.43	749.14	2.405	0.549
ADG, kg/d	1.458	1.477	1.463	0.014	0.504
Cold carcass weight, kg	448.48	446.67	447.43	2.021	0.816
Dressing percentage, %	59.58	59.45	59.72	0.002	0.669
Carcass SEUROP ^1^					
% of carcass conformation U	23.81	19.05	9.52	0.119	0.475
% of carcass conformation E	76.19	80.95	90.48	0.119	0.475
% of carcass fatness 2	33.33	28.57	23.81	0.143	0.800
% of carcass fatness 3	66.67	71.43	76.19	0.143	0.800

SS—sodium selenite; SY—vselenium-enriched yeast; OH-SeMet—hydroxy-selenomethionine. BW—body weight; ADG—average daily gain; ^1^ U—very good: profiles on the whole convex, very good muscle development; E—excellent: all profiles convex to super-convex, exceptional muscle development; 2—slight: slight fat cover, flesh visible almost everywhere; 3—medium important: flesh, with the exception of the round and shoulder, almost everywhere covered by fat, slight fat deposits in the thoracic cavity.

**Table 3:** Least square means pertaining to the effect of the selenium source during the finishing phase on the chemical composition, selenium content, shear force, thawing, drip, and cooking losses of the meat.

Item	Groups			SEM	*p*
	SS	SY	OH-SeMet		
Humidity, %	73.33	73.27	73.80	0.250	0.088
Crude Proteins, %	22.33	22.37	22.49	0.238	0.337
Ether extract, %	3.34	3.36	3.43	0.111	0.200
Ash, %	1.00	1.00	1.01	0.016	0.680
Selenium, mg kg^−1^ dry matter	0.342 ^a^	0.373 ^b^	0.426 ^c^	2.707	<0.01
Thawing loss, %	0.631	0.618	0.594	0.000	0.302
Drip loss, % after 8 days of storage	3.086 ^a^	2.226 ^b^	1.963 ^b^	0.001	<0.001
Cooking loss, %	30.19	29.83	29.89	0.006	0.836
Shear force, kg	3.067 ^a^	2.781 ^b^	2.753 ^b^	0.051	<0.0001

SS—sodium selenite; SY—selenium-enriched yeast; OH-SeMet—hydroxy-selenomethionine. Mean values with dissimilar letters were statistically different (*p* < 0.05).

**Table 4:** Least square means pertaining to the effect of the selenium source during the finishing phase on the pH of the meat during 8 days of storage.

Days of Storage	SS	SY	OH-SeMet	SEM
Day 1	5.70 ^a^	5.69 ^a^	5.70 ^a^	0.01
Day 2	5.70 ^a^	5.71 ^a^	5.71 ^a^	0.01
Day 3	5.71 ^a^	5.71 ^a^	5.71 ^a^	0.01
Day 4	5.74 ^b^	5.73 ^b^	5.74 ^b^	0.01
Day 5	5.83 ^c,x^	5.74 ^bc,y^	5.74 ^bc,y^	0.01
Day 6	5.88 ^d,x^	5.77 ^c,y^	5.76 ^c,y^	0.01
Day 7	5.94 ^e,x^	5.88 ^d,y^	5.87 ^d,y^	0.01
Day 8	6.03 ^f,x^	5.92 ^e,y^	5.91 ^e,y^	0.01
P(s) ^1^	0.0008			
P(d) ^1^	<0.0001			
P(s*d) ^1^	<0.0001			

SS—sodium selenite; SY—selenium-enriched yeast; OH-SeMet—hydroxy-selenomethionine. ^1^ s = selenium source; d = day of storage; s*d= selenium source ∗ day of storage. Means within a row without a common superscript differ; (x, y, *p* < 001). Means within a column without a common superscript differ (a, b, *p* < 0.001).

## Data Availability

The data presented in this study are available on request from the corresponding author.
